# Obstructive Fibrinous Tracheal Pseudomembrane Following Short‐Term Intubation: A Case Report and Management Considerations

**DOI:** 10.1155/crot/1913505

**Published:** 2026-10-09

**Authors:** Sami Khoury, Kevin Fung, Peng You, Julie Strychowsky

**Affiliations:** ^1^ Department of Otolaryngology-Head and Neck Surgery Schulich School of Medicine & Dentistry Western University London Ontario Canada uwo.ca

## Abstract

**Introduction:**

Obstructive fibrinous tracheal pseudomembrane (OFTP) is a rare but serious complication of endotracheal intubation that can cause airway obstruction, postextubation stridor, and respiratory distress. Due to its nonspecific presentation, it is often misdiagnosed and early recognition and intervention are critical to prevent respiratory compromise.

**Case:**

We present the case of a 17‐year‐old female who required 36 h of intubation for status epilepticus. She developed stridor within 24 h of extubation and presented with subglottic narrowing that did not respond to medical treatment. Bronchoscopy revealed a fibrinous pseudomembrane coating the subglottic and tracheal mucosa down to the carina, which was successfully removed, leading to complete symptom resolution. She did not have any recurrence postoperatively and no residual symptoms at her follow‐up.

**Discussion:**

This case highlights the importance of considering OFTP in patients with postextubation stridor refractory to medical therapy. While ischemic injury from high endotracheal cuff pressures is a known cause, mechanical trauma and aspiration may also contribute. Bronchoscopy is essential in the diagnosis and treatment of this condition.

## 1. Introduction

Obstructive fibrinous tracheal pseudomembrane (OFTP) is a rare but potentially life‐threatening complication of endotracheal intubation in which a fibrinous membrane can obstruct the airway [1, 2]. Its nonspecific presentation can delay recognition. We report OFTP in an adolescent after 36 h of intubation to highlight its consideration in postextubation stridor refractory to medical therapy and to document the clinical course after pseudomembrane removal.

## 2. Case Presentation

A 17‐year‐old girl (168.3 cm, 122 kg) presented to a community hospital emergency department with generalized tonic–clonic movements, eye fluttering, and eyes rolling upwards. She had a history of psychogenic nonepileptic seizures and epilepsy, with no prior history of upper or lower airway disease and no previous intubation or tracheostomies. Despite receiving midazolam, levetiracetam, and propofol, her condition did not improve, necessitating intubation. She was premedicated with propofol and rocuronium and was intubated with a MAC 3 blade and a 7.5‐mm cuffed endotracheal tube (Grade 1 view, first attempt, no trauma); based on her body habitus, a 7.0 or 7.5 mm internal diameter tube would have been size appropriate and cuff pressures were not documented in the medical record. She was then transferred to the pediatric critical care unit at a tertiary care academic Children’s Hospital.

Following transport, phenytoin was administered, and the abnormal movements ceased. Tube position was confirmed to be appropriate on postintubation imaging. Further ventilatory parameters, including the mode of ventilation or whether the tube had moved could not be determined from the medical records. Sedation was weaned over 24 h, and a CT head showed no new findings. She was extubated 36 h postintubation, requiring 1‐2 L oxygen, which was gradually weaned to room air. Extubation was uneventful, and she was discharged 3 days later on an increased antiseizure medication dose.

Within 24 h, she developed increased work of breathing and stridor, prompting representation to the community emergency room. A CT neck showed mild subglottic narrowing (Figure 1). She was transferred again for further assessment. Flexible nasopharyngolaryngoscopy confirmed narrowing at the subglottis (Figure 2), initially attributed to inflammation or granulation tissue. With mild stridor and no work of breathing, she was treated with dexamethasone, nebulized ciprofloxacin, and dexamethasone and nebulized epinephrine as needed and admitted for observation. Inflammatory markers were mildly elevated (C‐reactive protein of 86 mg/L and normal WBC).

**FIGURE 1:**
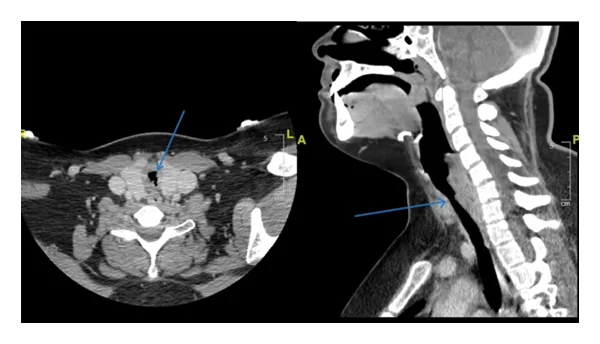
CT neck with contrast highlighting the area of subglottic narrowing. Blue arrows point to the areas of narrowing on both views.

**FIGURE 2:**
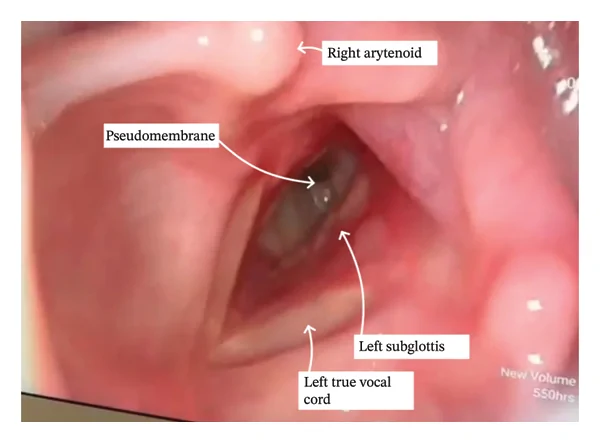
Flexible nasopharyngolaryngoscopic view of the larynx. Labels and white arrows identify the right arytenoid, left true vocal cord, left subglottis, and pseudomembrane within the subglottic airway. The pseudomembrane narrows the airway below the true vocal cords.

Over 48 h, her stridor persisted, and nasopharyngolaryngoscopy continued to show significant subglottic narrowing. Two episodes of increased work of breathing responded to nebulized epinephrine and salbutamol. She was taken to the operating room for a diagnostic laryngoscopy and bronchoscopy.

In the operating room, under spontaneous ventilation, a Dedo laryngoscope provided a Grade 1 view, and she was placed into laryngeal suspension. The supraglottis and glottis were normal. The subglottis and trachea had a nearly circumferential gelatinous inflammatory pseudomembrane, which was removed using microlaryngeal suction and cup forceps, revealing healthy underlying mucosa (Figures 3 and 4). Postoperatively, she was monitored in the pediatric critical care unit. Within 24 h, her stridor completely resolved, and she remained asymptomatic and without respiratory distress.

**FIGURE 3:**
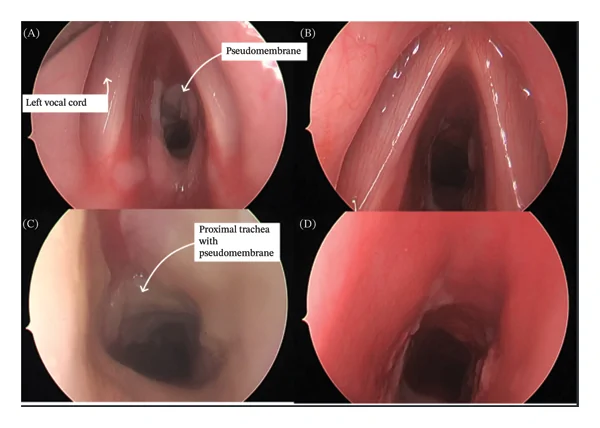
Intraoperative direct laryngoscopy and bronchoscopy before and after pseudomembrane removal. (A) Glottic view before intervention, with labels and white arrows identifying the left vocal cord and subglottic pseudomembrane. (B) Glottic and subglottic view after pseudomembrane removal. (C) Proximal trachea before intervention, with a label and white arrow identifying the pseudomembrane coating the tracheal wall. (D) Proximal trachea after removal, showing the patent airway and exposed underlying mucosa.

**FIGURE 4:**
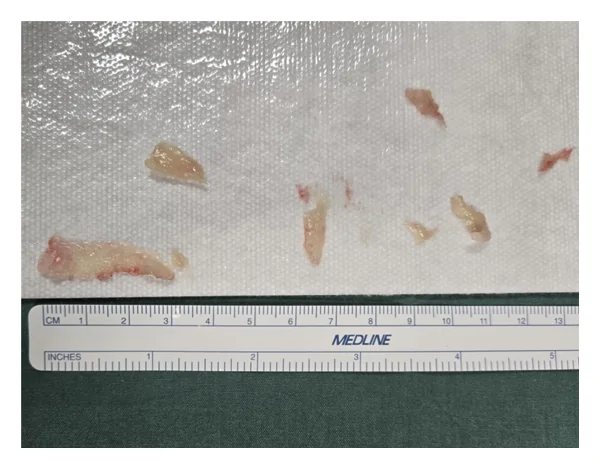
Appearance of the pseudomembranes extracted measuring up to 3.0 cm. Final pathology came back as “fibrin with entrapped inflammatory cells; negative for malignancy.”

Histopathologic examination of the excised pseudomembrane showed fibrin with entrapped inflammatory cells and no evidence of malignancy. Bacterial tissue culture of the pseudomembrane obtained intraoperatively grew *Rothia mucilaginosa*, *Streptococcus mitis* group, *Actinomyces* species, and *Eikenella corrodens*. The mixed growth was considered oral flora rather than evidence of a causative bacterial infection. No bacterial cultures of sputum or respiratory secretions, fungal cultures, or investigations for viral pathogens were performed.

She was discharged from hospital once her respiratory status had stabilized. She represented 3 weeks later with increased work of breathing; flexible endoscopic evaluation at that time demonstrated paradoxical vocal fold motion without recurrence of the pseudomembrane. Given her history, she underwent a repeat diagnostic laryngoscopy and bronchoscopy under general anesthesia, which showed a completely normal airway with no evidence of recurrent pseudomembrane. She was subsequently seen in clinic approximately 1 month later, at which time she had a normal voice, no exertional dyspnea, and no new endoscopic findings. She has had no further hospital presentations for airway complaints in the 14 months since her last diagnostic laryngoscopy and bronchoscopy.

## 3. Discussion

OFTP is an uncommon but significant cause of postextubation airway obstruction that can mimic laryngeal edema, tracheomalacia, or postintubation subglottic stenosis. Although OFTP itself has been previously described, this case highlights the diagnostic significance of early recognition of this condition even without classically associated risk factors. This patient developed nearly circumferential pseudomembranes despite a relatively short intubation, a documented Grade 1 atraumatic first pass attempt with a sized 7.5‐mm tube. This case illustrates that OFTP should remain on the differential for refractory postextubation stridor even when classically described risk factors are absent or unconfirmed.

The pathophysiology of OFTP remains debated. One mechanism involves initial traumatic intubation leading to desquamation of the subglottic mucosa. The subglottis is especially vulnerable as it is completely surrounded by cricoid cartilage. This is in keeping with the fact that the most common site for OFTP is at the level of the subglottis [3, 4]. However, in many cases including this case, the pseudomembrane extends past the subglottis down to the level of the carina [2, 5]. Another mechanism involves ischemic injury from prolonged or excessive endotracheal tube cuff pressure leading to mucosal necrosis and fibrin deposition. However, patients can develop OFTP with uncuffed endotracheal tubes [2] and usually present after a short‐term intubation with an average of 36 h [6]. The brief duration of intubation and absence of reported trauma do not establish the mechanism of pseudomembrane formation or exclude ischemic injury. Cuff pressure was not documented for this patient and cannot be confirmed to be a contributing factor. Although she was not fasted prior to emergency intubation, there was no objective clinical evidence of aspiration such as witnessed vomiting and gastric contents on suctioning or supportive imaging findings. These findings suggest that the etiologic factors classically implicated in OFTP were not clearly operative in this patient and that additional unidentified mechanisms may underlie some cases of this condition.

The clinical presentation of OFTP varies, but the most common symptom is stridor, which typically occurs within hours to days after extubation. Diagnostic imaging, including CT scans, can provide clues but are often insufficient for definitive diagnosis. Bronchoscopy remains the gold standard for visualizing the pseudomembrane and confirming the diagnosis. In this case, nasopharyngolaryngoscopy suggested subglottic narrowing, but it was only during microdirect laryngoscopy and bronchoscopy that the fibrinous membrane was definitively identified and removed. The endoscopic and histopathologic findings supported OFTP; however, fibrin with inflammatory cells is not sufficient to exclude an infectious cause. This underscores the importance of early bronchoscopy in patients with unexplained postextubation stridor to avoid delays in diagnosis and treatment.

Infectious pseudomembranous tracheitis is an important differential diagnosis because bacterial, fungal, and viral infections can also cause airway pseudomembranes [7]. In this patient, the mixed bacterial growth from pseudomembrane tissue was considered oral flora. The diagnosis of postintubation OFTP was supported by the temporal relationship to intubation, endoscopic appearance, histopathology, and clinical course, in addition to microbiology ruling out any pathological organisms.

Management of OFTP primarily involves mechanical removal of the pseudomembrane, most commonly via rigid or flexible bronchoscopy. The rapid resolution of symptoms following the procedure further supports the diagnosis of OFTP. Previous case reports have shown that timely intervention leads to excellent outcomes, with low recurrence rates and minimal long‐term complications [5, 6]. However, mortality remains significant, reported at 3.7% [6], highlighting the need for prompt diagnosis and treatment. Early bronchoscopy should be considered in patients with postextubation stridor that do not respond to corticosteroids or nebulized epinephrine, is persistent or recurrent, or is accompanied by worrisome imaging or endoscopic findings.

The patient’s subsequent episode of respiratory difficulty was attributed to paradoxical vocal fold motion, with no recurrent pseudomembrane on repeat endoscopy. Her return to normal voice and breathing at outpatient follow‐up and absence of further airway complaints over 14 months provide longer‐term outcome data supporting sustained recovery after removal.

## 4. Conclusion

OFTP is a rare but serious complication of endotracheal intubation that can lead to life‐threatening airway obstruction if not promptly diagnosed and managed. This case report highlights that OFTP can occur after brief intubation when classically described risk factors are absent or unconfirmed. Early bronchoscopy should be considered in patients with unexplained postextubation stridor especially when there is a poor response to medical interventions. In this case, mechanical removal of the pseudomembranes led to rapid symptom resolution and the patient remained recurrence free at 14 months of follow‐up.

## Funding

There was no funding for this project.

## Consent

Consent was obtained from this patient to take pictures and to use them for publication.

## Conflicts of Interest

The authors declare no conflicts of interest.

## Data Availability

Data sharing is not applicable to this article as no new data were created or analyzed in this study.
